# Characterization of *Aspergillus nidulans* DidB^Did2^, a non-essential component of the multivesicular body pathway

**DOI:** 10.1016/j.fgb.2010.03.010

**Published:** 2010-07

**Authors:** América Hervás-Aguilar, Olga Rodríguez-Galán, Antonio Galindo, Juan F. Abenza, Herbert N. Arst, Miguel A. Peñalva

**Affiliations:** a Department of Molecular and Cellular Medicine. Centro de Investigaciones Biológicas CSIC, Ramiro de Maeztu 9, Madrid 28040, Spain; b Department of Microbiology, Imperial College London, Flowers Building, Armstrong Road, London SW7 2AZ, UK; c Department of Molecular Biosciences, University of Kansas, Lawrence, KS 66045, USA

**Keywords:** Membrane traffic, Endosomes, Multivesicular body pathway, *Aspergillus nidulans*

## Abstract

ESCRT-III heteropolymers mediate membrane protein cargo sorting into multivesicular endosomes for subsequent vacuolar degradation. We studied the localization of largely uncharacterized *Aspergillus nidulans* ESCRT-III using its key structural component Vps32 and the ‘associated’ component DidB^Did2^. Vps32-GFP localizes to motile early endosomes as reported, but predominates in aggregates often associated with vacuoles due to inability to dissociate from endosomes. DidB^Did^*^2^* regulating Vps4 (the ATPase disassembling ESCRT-III) is not essential. Consistent with this accessory role, *didB*Δ is unable to block the MVB sorting of the glutamate transporter AgtA, but increases its steady-state level and mislocalizes a fraction of the permease to the plasma membrane under conditions promoting its vacuolar targeting. *didB*Δ exacerbates the dominant-negative growth defect resulting from Vps32-GFP over-expression. A proportion of DidB-GFP is detectable in early endosomes colocalizing with RabA^Rab5^ and accumulating in *nudA1* tips, suggesting that ESCRT-III assembles on endosomes from the early steps of the endocytic pathway.

## 1 Introduction

The understanding of the physiological roles of the ESCRT (endosomal sorting complex required for transport) machinery at the molecular level has received considerable impetus over the last few years. The ESCRT machinery plays roles in membrane abscission during metazoan cell division (Morita et al., 2007; Carlton and Martín-Serrano, 2007; Yang et al., 2008) and in the budding of enveloped viruses from the plasma membrane but was originally discovered for its fundamental role in cargo sorting and inward endosomal membrane budding during the formation of multivesicular endosomes (Katzmann et al., 2001; Katzmann et al., 2002; Babst et al., 2002a; Babst et al., 2002b; Katzmann et al., 2003; Williams and Urbe, 2007).

In ascomycete fungi, ESCRT-I, II and certain ESCRT-III proteins play an additional positive role in ambient pH signal transduction, as they are non-dedicated components of the signal transduction pathway that mediates activation of the PacC/Rim101 transcription factors regulating changes in gene expression in response to alkaline pH (Xu and Mitchell, 2001; Vincent et al., 2003; Xu et al., 2004; Kullas et al., 2004; Hayashi et al., 2005; Rothfels et al., 2005; Cornet et al., 2005; Blanchin-Roland et al., 2005; Boysen and Mitchell, 2006; Galindo et al., 2007; Blanchin-Roland et al., 2008; Mitchell, 2008; Rodríguez-Galán et al., 2009; Herrador et al., 2009). In *Aspergillus nidulans*, however, investigation of the physiological roles of ESCRT-III has been hampered by the lack of availability of classical mutations, which reflects the facts that genes encoding the key components of the ESCRT machinery are essential or nearly so (Calcagno-Pizarelli, A.M., M.A.P and H.N.A., unpublished) (Rodríguez-Galán et al., 2009) and that the consequences of partial deficiencies are unknown, precluding the design of rational screens.

There are six denoted ESCRT-III-like proteins in *S. cerevisiae*: the key ESCRT-III elements Vps20p, Vps32p, Vps2p, Vps24p and the accessory factors Did2p and Vps60p. Vps32, the major structural component of ESCRT-III, oligomerizes on endosomal membranes, most likely driving membrane invagination. Vps32 oligomerization is initiated by Vps20 and terminated by Vps24, which ‘caps’ Vps32 oligomers. Vps24 recruits Vps2 which, in concert with accessory factors like Did2 recruits the AAA ATPase Vps4 that disassembles the complex and releases its components to the cytosol for new cycles of sorting/budding (Hanson et al., 2008; Teis et al., 2008; Saksena et al., 2009). In agreement, the budding reaction has been reconstituted *in vitro* and used to demonstrate that Vps20, Vps32 and Vps24 suffice to form multivesicular bodies, whereas neither Vps4 itself nor Vps2, the major recruiter of Vps4 (Obita et al., 2007), are required for one round of vesicle budding, although they are required for initiating new rounds of vesicle formation (Wollert et al., 2009). The ESCRT-III-associated elements include, in addition to ESCRT-III-like Did2p and Vps60p, two factors denoted Vta1p and Ist1p (for clarity we use the *Saccharomyces cerevisiae* nomenclature throughout, unless otherwise indicated). These ESCRT-III associated proteins regulate and coordinate Vps4p activity with ESCRT-III disassembly (Amerik et al., 2000; Lottridge et al., 2006; Nickerson et al., 2006; Azmi et al., 2006; Azmi et al., 2008; Dimaano et al., 2008; Rue et al., 2008). Null alleles of these genes lead to weak multivesicular body sorting phenotypes in *S. cerevisiae*. Thus we anticipated that the corresponding mutations in *A. nidulans* might not be lethal.

All six ESCRT-III-like proteins contain a basic N-terminal four-helical core, which binds membranes (Lin et al., 2005; Obita et al., 2007), and an acidic C-terminal region that binds regulatory factors, such as the Vps4p ATPase [in the cases of Vps2p and Did2p (Obita et al., 2007)] or the endosomal protein ALIX (Bro1p in yeast) [in the case of the mammalian Vps32 homologue CHMP4 (McCullough et al., 2008)]. ESCRT-III-like proteins are held in a closed conformation through autoinhibitory intramolecular interaction between the basic and acidic regions, which allows tight control of ESCRT-III assembly (Zamborlini et al., 2006). Due to these facts, ESCRT-III proteins are functionally inactivated by C-terminal attachment of bulky tags such as GFP/RFP. Notably, fluorescent protein tagging does not however interfere with their localization to membranes, because the latter is mediated by the N-terminal domain (Lin et al., 2005; Muziol et al., 2006; Nickerson et al., 2006).

In the filamentous fungi *Ustilago maydis* and *Aspergillus nidulans*, early endosomes are readily recognizable because they display bidirectional motility on microtubule (MT) tracks (Wedlich-Soldner et al., 2002; Peñalva, 2005; Lenz et al., 2006; Steinberg, 2007; Zekert and Fischer, 2008; Abenza et al., 2009). Early endosomes travel to the tip, towards which the plus-end of MTs is oriented, in a kinesin-dependent manner before being loaded on dynein and reversing the direction of their movement, which led Steinberg and co-workers (Lenz et al., 2006) to coin the term ‘dynein loading zone’ to denote the tip region where endosomes shift their motors, which is conserved in *A. nidulans* (Zekert and Fischer, 2008; Abenza et al., 2009). Thus, due to the relative ease with which early endosomes can be recognized, these organisms are ideally suited to study endosomal related processes. Moreover, an additional identity landmark of these fungal early endosomes is that they accumulate in large aggregates in the tip region when dynein function is deficient (Zekert and Fischer, 2008; Abenza et al., 2009). Finally, long-distance bidirectional movement is very different from the short distance oscillatory movement of Golgi equivalents and ER exit sites (Pantazopoulou and Peñalva, 2009), further underscoring its diagnostic value.

We show here that the *A. nidulans DID2* orthologue denoted *didB* is indeed not essential, report a detailed characterization of the subcellular localization of Vps32 and exploit the characteristic motility and trafficking of early endosomes to demonstrate that these contain a proportion of DidB.

## 2 Materials and methods

### 2.1 *Aspergillus nidulans* techniques

*A. nidulans* strains, whose genotypes are described in Table 1, carried markers in standard use (Clutterbuck, 1993; Nayak et al., 2005; Calcagno-Pizarelli et al., 2007). Phenotype testing using plate tests diagnostic of pH regulatory functions (Tilburn et al., 1995; Peñas et al., 2007) and mycelial culture conditions for constant pH and pH shift experiments were as described (Hervás-Aguilar et al., 2007). All recombinant strains constructed by transformation were confirmed to carry the expected single-copy integration events by Southern analysis with appropriate probes. Epitope-tagged transgenes segregating in meiotic crosses were genotyped by PCR, using primer pairs flanking the epitope(s) coding regions.

### 2.2 Recombinant strains

A plasmid driving expression of Vps32-mRFP under the control of the *vps32* promoter (our collection No. p1731) was constructed as follows: A DNA fragment containing the complete *vps32* coding region preceded by the 600 bps upstream of the initiation ATG was amplified by PCR. This fragment was combined using fusion PCR with a cassette containing a (Gly-Ala)5 linker followed by the coding region of mRFP and the *A. fumigatus pyrG* gene, such that the C-terminus of Vps32 was fused in frame to the (Gly-Ala)5 linker followed by the mRFP coding region (De Souza et al., 2004; Yang et al., 2004). The resulting fragment was cloned into pCR2.2 Topo to yield p1731, that was used for transformation (Tilburn et al., 1983). Transformants were analyzed by Southern analyses and one clone carrying a single integration event of the transforming plasmid into the *vps32* locus was selected for microscopy. A strain in which the *didB* gene had been replaced by *didB-gfp* encoding a C-terminal fusion between DidB and GFP were constructed by transformation. The *didB-gfp* transformation cassette was constructed as reported (Yang et al., 2004). The *didBΔ* null allele was constructed by homologous recombination following transformation with a linear DNA fragment, constructed by conventional cloning, in which the complete coding region of *didB* had been replaced by the *A. fumigatus pyroA* gene (*pyroA^Af^*), which was flanked by ∼700 and 600 bps of *didB* upstream and downstream flanking regions, respectively. A strain carrying the gene replacement event was identified after Southern analysis. In a number of meiotic crosses the growth phenotype of *didBΔ* co-segregated with the deletion construct, which was detected by diagnostic PCR, strongly indicating the absence of second-site mutations originated during the transformation procedure that contribute to the phenotype.

### 2.3 GST-pull down assay

This was carried out essentially as described (Galindo et al., 2007; Rodríguez-Galán et al., 2009), with the following modification: the presence of the protein A IgG-binding ZZ domain in the N-terminus of the Vps32 and Vps4 preys allows sensitive detection of the preys using western blots. GST-pull downs of bacterial extracts expressing GST baits were mixed with bacterial extracts expressing the preys. After incubation at 4 °C in 10 mM Tris–HCl pH 8.0, 1 mM EDTA, 5 mM DTT, 0.5% Triton-X-100 and 200 mM NaCl, GST fusion proteins and bound material were pulled-down using glutathione-sepharose beads. Pulled-down proteins were loaded onto duplicate 10% polyacrylamide gels. One replica was stained with Coomassie blue, whereas the second replica was blotted onto a nitrocellulose membrane that was first reacted with mouse anti-Myc mouse IgG (1/500) and subsequently incubated with peroxidase-coupled sheep anti-mouse IgG (Amersham, 1/10,000). ZZ domain-containing proteins were detected by chemiluminescence.

### 2.4 Microscopy

‘Watch minimal medium’ (WMM) used to culture cells for microscopy and FM4-64 loading have been described (Peñalva, 2005). We used an incubation temperature of 25–27 °C. For studies on AgtA-GFP endocytic downregulation, cells were pre-cultured for 16 h on WMM containing 5 mM ammonium tartrate and 0.5% glucose as sole nitrogen and carbon sources, respectively, transferred to the same medium in which ammonium tartrate was replaced by 5 mM l-glutamate and incubated for a further 2 h to allow synthesis of the AgtA transporter. Endocytic downregulation of the transporter (Apostolaki et al., 2009) was triggered after subsequent transfer of cells incubated in glutamate medium to medium containing 5 mM ammonium tartrate. Cells were photographed before and 30 min after the shift from glutamate-containing to ammonium-containing medium.

For studies on the subcellular localization of Vps32-mRFP expressed at physiological levels, cells were cultured on WMM containing 0.1% glucose (w/v) and 5 mM ammonium tartrate. Very high levels of expression of Vps32-GFP driven by the *alcA^p^* were achieved by pre-culturing the cells on WMM containing 0.02% glucose (w/v) before transferring the cells to the same medium containing 1% ethanol and subsequent incubation for a further 3 h. Relatively moderate expression of Vps32-GFP was achieved by culturing cells on WMM containing 0.1% fructose (w/v) as sole carbon source. These conditions are non-inducing but also non-repressing for the *alcA^p^* (Felenbok et al., 2001).

For most experiments we used an upright Nikon Eclipse 80i microscope equipped with Semrock Brightline GFP-3035B and TXRED-4040B filters for GFP and mRFP/FM4-64 fluorescence, respectively. The microscope was equipped with a 100-W mercury lamp epifuorescence module, a Uniblitz external shutter and ×100 1.40 or ×60 1.40 NA plan apochromat objectives. Image acquisition was carried out with a Hamamatsu ORCA-ER camera driven by METAMORPH (Molecular Devices, USA). Cells were cultured attached to microscope coverslips as described (Peñalva, 2005). Coverslips were mounted on microscopy slides.

For experiments involving motile early endosomes decorated with DidB-GFP we used four-chambered Lab-Tek coverglasses and a Leica DMI6000B inverted microscope with motorized z-focus, equipped with a Leica EL6000 external light source for epifluorescence excitation and a Semrock Brightline GFP-3035B filter set. This microscope was also driven by METAMORPH (Molecular Devices) software using a DMI6000-specific driver. Images were acquired using HCX ×63 1.4 numerical aperture (NA) and a Hamamatsu ORCA ER-II cooled charge-coupled device (CCD) camera. Colocalization experiments with mCherry-RabA and DidB-GFP were made using a DualView beam splitter, as described (Pantazopoulou and Peñalva, 2009). Kymographs, linescan plots and movie manipulation were carried out using METAMORPH and ImageJ (http://rsbweb.nih.gov/ij/).

### 2.5 Western blots

*A. nidulans* extracts, SDS–PAGE and western blotting were made as described (Calcagno-Pizarelli et al., 2007; Hervás-Aguilar et al., 2007). Nitrocellulose blots were reacted with Roche’s anti-GFP cocktail of two mouse monoclonal antibodies (clones 7.1 and 13.1) (1/5000) in combination with peroxidase-coupled sheep anti-mouse IgG (Amersham, 1/4000) as secondary antibody. Peroxidase activity was detected with ECL (Amersham).

## 3 Results

### 3.1 Vps4 interacts directly with DidB as determined by pull-down assays

The *A. nidulans* orthologue of *S. cerevisiae DID2* is denoted *didB* and corresponds to AN9396.4 (http://www.aspgd.org). We confirmed the position of the predicted single intron by cDNA sequencing (not shown). DidB, which has 209 residues, shows 30.8% amino acid sequence identity to its 204 residue *S. cerevisiae* orthologue. Did2p contains a short C-terminal motif, denoted MIM1, that mediates binding to the MIT domain in the Vps4 AAA ATPase with micromolar affinity (Nickerson et al., 2006; Stuchell-Brereton et al., 2007; Obita et al., 2007). The MIM1 motif consensus sequence (D/E)xxLxxRLxxL(K/R) (where x indicates any amino acid) is fully conserved in the C-terminus of DidB, as would be expected if it binds *A. nidulans* Vps4.

**Fig. 1:**
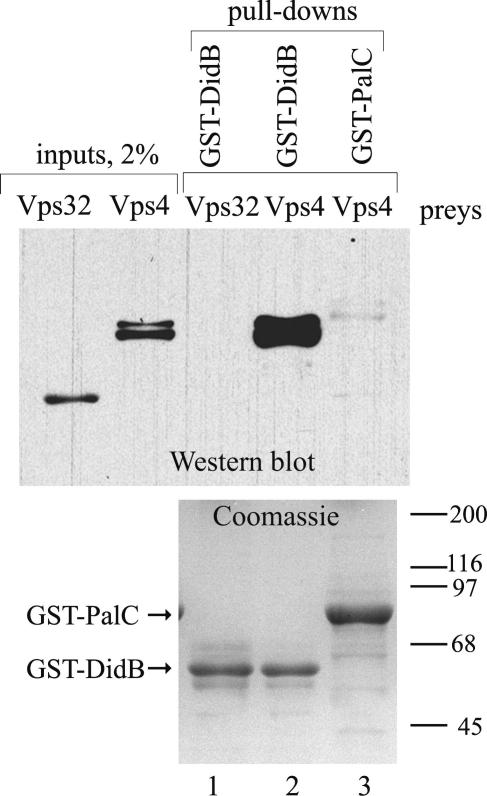
GST-DidB specifically pulls-down Vps4. N-terminal fusion proteins between the ZZ (IgG-binding) domain of protein A and Vps32 or Vps4 were expressed in *E. coli* and used in pull-down assays with GST-DidB or GST-PalC fusion protein baits. GST-DidB pulled down Vps4 but not Vps32. The upper panel shows a western blot analysis of ZZ domain-containing proteins. The lower panel shows a Coomassie staining of the GST fusion protein baits used for pull-downs.

We have previously determined that DidB interacts with Vps4 in yeast two-hybrid assays (Rodríguez-Galán et al., 2009). However, ESCRT-III proteins are highly conserved and thus the possibility that Vps4 and DidB interact indirectly in two-hybrid experiments, using another ESCRT-III protein from *S. cerevisiae* to bridge their interaction, could not be ruled out. Thus we carried out GST pull-down assays (Fig. 1), which demonstrated that bacterially expressed ZZ-Vps4 but not ZZ-Vps32 copurifies with bacterially expressed GST-DidB on glutathione-sepharose beads. ZZ-Vps4 does not co-purify with the unrelated bait GST-PalC, which we used as negative control. Thus, *A. nidulans* DidB, like its *S. cerevisiae* Did2p and human CHMP1 orthologues, interacts directly with Vps4. These data support the contention that DidB and *S. cerevisiae* Did2p share a conserved function in coordinating the disassembly of ESCRT-III polymers from endosomal membranes.

### 3.2 DidB is not essential but its absence impairs the downregulation by endocytosis of a plasma membrane transporter

**Fig. 2:**
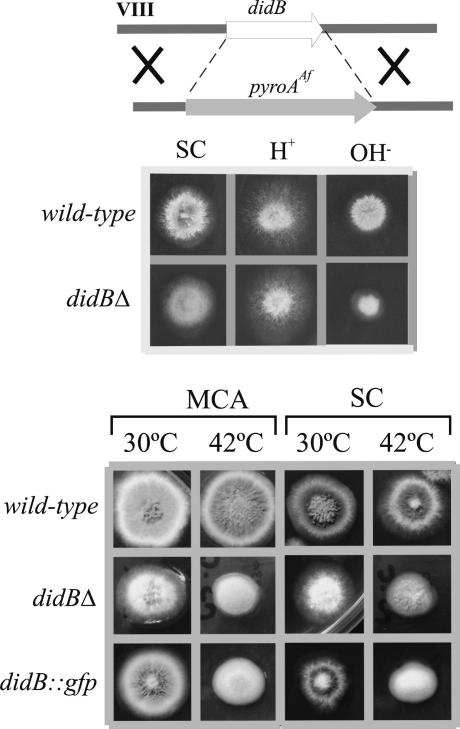
Phenotypic characterization of *didBΔ*. Top: schematic representation of the gene replacement procedure. Middle: growth and alkaline pH sensitivity. Strains were tested for growth at 37 °C on synthetic complete medium (SC) and on SC containing 0.5 M NaH_2_PO_4_ (acidic medium, H^+^) or 0.2 M Na_2_H PO_4_ (∼pH 8, OH^−^), as indicated. Bottom: Strains were cultured at 30 °C or 42 °C for 3 days on SC or on *Aspergillus* complete medium (MCA).

We deleted the complete *didB* ORF by homologous recombination (Fig. 2A). In agreement with our prediction that *didBΔ* would have a less severe phenotype than the nearly lethal phenotype resulting from deletion of genes encoding ‘core’ ESCRT components (Calcagno-Pizarelli, A.M., M.A.P and H.N.A., unpublished) (Rodríguez-Galán et al., 2009), *didBΔ* strains are viable, although they show impaired growth and conidiation. Such impairment is weak at 37 °C but more conspicuous at 42 °C. At 37 °C *didBΔ* also results in weak alkaline pH sensitivity (Fig. 2). Partial loss-of-function mutations in the PacC-dependent pH regulatory system also result in weak alkaline pH sensitivity (Tilburn et al., 2005; Peñas et al., 2007; Calcagno-Pizarelli et al., 2007) but western blot analysis demonstrated that *didBΔ* does not impair the proteolytic processing activation of PacC (data not shown), establishing that this phenotype is unrelated to ambient pH signaling. As deletion of a large number of *S. cerevisiae* genes involved in vacuolar and endosomal biogenesis also results in alkaline pH sensitivity (Serrano et al., 2004), this *didBΔ* phenotype would be consistent with weak impairment of *A. nidulans* endosome/vacuole biogenesis, as expected from its predicted role.

We tested the effects of *didBΔ* in endocytosis. Using FM4-64 (Peñalva, 2005), we determined that the mutation does not detectably affect either bulk membrane internalization or vacuolar biogenesis. *S. cerevisiae* null mutations in 15 ‘class E’ genes encoding ESCRT-0, I, II and III subunits and the Vps4 ATPase result in formation of large, abnormal endosomal structures adjacent to the vacuoles, denoted class E compartments (Bowers and Stevens, 2005). *did2Δ* leads to a weak class E phenotype (Nickerson et al., 2006; Rue et al., 2008). However, no membrane aggregates which might resemble class E compartments were seen adjacent to *didBΔ* mutant vacuoles (data not shown).

**Fig. 3:**
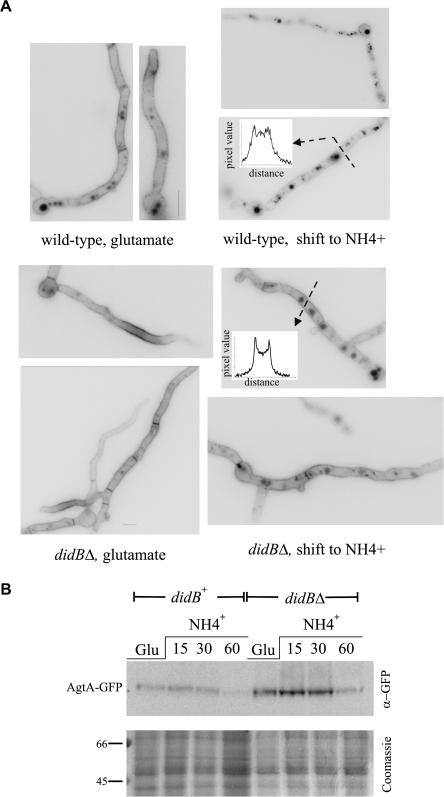
*didBΔ* impairs the endocytic downregulation of the dicarboxylic amino acid permease AgtA. (A) Wild-type or *didBΔ* cells carrying *agtA-GFP* were incubated for 2 h in the presence of glutamate as sole nitrogen source, which results in expression of AgtA (left panels). After this period, cells were transferred to medium containing 10 mM ammonium tartrate as sole nitrogen source and incubated for a further 30 min before being photographed (right panels). Note the very faint staining of the plasma membrane seen in the wild-type after incubation in the presence of ammonium and the more prominent staining of the plasma membrane in the *didBΔ* mutant strain, as illustrated by the linescans shown on the right of the respective panels, which were taken across the indicated regions. The pixel values in the y axes represent arbitrary units. (B) Total membrane fractions (including endosomal membranes in addition to plasma membrane) were isolated from *agtA-GFP* cells cultured on glutamate (Glu) or from cells cultured on glutamate and shifted to NH4+ for the indicated time periods. Membrane proteins were solubilized in Laemmli loading buffer and analyzed by western, using an α-GFP antibody. A replica of the gel was stained with Coomassie to confirm approximately equal loading. Shown is the region of the gel corresponding to 45 through 66 kDa.

We next determined the effects of *didBΔ* in the ammonium-dependent vacuolar sorting of the dicarboxylic amino acid permease AgtA, a demonstrated endocytic cargo of the multivesicular body pathway (Apostolaki et al., 2009). In cells cultured on glutamate, AgtA, endogenously tagged with GFP in its C-terminus, localizes to the plasma membrane and to the vacuolar lumen (the latter localization reflecting the normal turnover of the permease), as described (Apostolaki et al., 2009). *agtA* transcription is very sensitive to nitrogen metabolite repression. Ammonium also promotes the turnover of AgtA. Thus, if glutamate-cultured cells are shifted to medium containing ammonium, *agtA* transcription is shut off and plasma membrane-localized AgtA is internalized to endosomes, sorted into the multivesicular body pathway and delivered to the vacuolar lumen (Abenza et al., 2009; Apostolaki et al., 2009) (Fig. 3A, upper row). In contrast to the wild-type, AgtA-GFP almost exclusively localized to the plasma membrane in *didBΔ* cells cultured on glutamate because the labeling of vacuoles seen in the wild-type was markedly less conspicuous in the mutant (Fig. 3A, lower left), strongly indicating that delivery of AgtA to the vacuole is deficient. An additional difference between wild-type and *didBΔ* cells occurred after shifting cells to ammonium. Although *didBΔ* did not prevent the ability of AgtA to reach the vacuolar lumen as in the wild-type, it led to an increased proportion of the permease remaining in the plasma membrane (Fig. 3A, lower right; this difference is illustrated by the linescans shown on the right).

In *S. cerevisiae*, it is well established that mutations leading to relatively mild impairment of the multivesicular body pathway promote the recycling of the Gap1p amino acid permease from the membranes of late endosomes and vacuoles to the plasma membrane (Nikko et al., 2003; Nikko and Andre, 2007). This is relevant because Gap1p is, like AgtA, endocytically downregulated by ammonia. Thus, the abnormal localization of AgtA to the plasma membrane seen in *didBΔ* cells both on glutamate and after promoting its vacuolar targeting with ammonium would be consistent with mild impairment of the multivesicular body pathway.

Deficient sorting into the multivesicular body pathway should decrease the turnover of AgtA. In agreement, western blot analysis of membrane fractions showed that the steady-state level of AgtA-GFP is conspicuously higher in *didBΔ* than in wt cells before shifting cells to ammonia and remains so even at 60 min after promoting its endocytic internalization (Fig. 3B). At this time point, levels of AgtA in *didBΔ* were similar to those of the wt before the shift to ammonium. These data are consistent with epifluorescence observations. Thus, we conclude that *didBΔ* weakly affects the endocytic downregulation of AgtA at the level of endosomes.

### 3.3 The subcellular localization of *A. nidulans* Vps32/ESCRT-III

We next determined the endomembrane compartments on which ESCRT-III is assembled using Vps32 (the major component of ESCRT-III lattices) tagged with GFP. Vps32-GFP is targeted to endosomes through its N-terminal domain. As noted in the introduction, it is well established that C-terminal attachment of fluorescent proteins to Vps32 impedes its function (Howard et al., 2001; Strack et al., 2003; Lin et al., 2005; Nickerson et al., 2006; Zamborlini et al., 2006). Thus, the fact that *A. nidulans* *vps32* is virtually essential (Calcagno-Pizarelli, A.M., M.A.P and H.N.A., unpublished) precludes the construction of strains expressing endogenously tagged Vps32-GFP.

Thus, we first used a transgene expressing Vps32-GFP under the control of the inducible alcohol dehydrogenase gene promoter (*alcA*^p^). This transgene has been used previously in the context of the analysis of the pH signaling protein PalC (Galindo et al., 2007) to show, using non-induced (thus relatively low) levels of expression, that a proportion of Vps32 localizes to early endosomes, which are easily recognizable by their long-distance bidirectional movement (Peñalva, 2005; Abenza et al., 2009) (see Section 1).

**Fig. 4:**
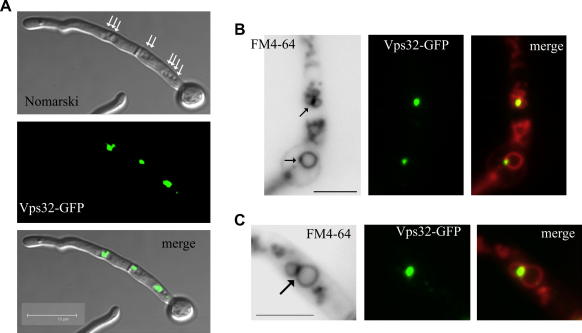
Forced expression of Vps32-GFP: subcellular localization. (A) Cells carrying an *alcA^p^*::*vps32-gfp* transgene were pre-cultured under repressing conditions and shifted to inducing conditions (WMM containing 1% v/v ethanol) for 3 h, which results in strong expression of the transgene. Cells were photographed using a confocal microscope in the Nomarski and GFP channels, as indicated. Note the large, strongly fluorescent aggregates of membranes containing Vps32-GFP adjacent to clusters of vacuoles, which are indicated by white arrows. The bar represents 10 μm. (B) and (C) Cells cultured under non-repressing and also non-inducing conditions (WMM containing 0.1% fructose as sole carbon source) were labeled with FM4-64 and observed by epifluorescence microscopy in the green and red channels (FM4-64, shown in inverted contrast for clarity), as indicated. Membrane aggregates containing Vps32-GFP that are also labeled by FM4-64 are indicated by arrows. Note that they are often associated with contact regions between adjacent vacuoles. Bars, 5 μm.

Fig. 4A displays new data obtained under conditions leading to strong over-expression of Vps32-GFP. In these experiments, cells were cultured under repressing conditions and shifted to inducing conditions for 3–4 h. Overexpressing hyphae did not have any cytosolic fluorescence and showed instead strong accumulation of fluorescence in large accretions adjacent to the vacuoles. These accretions most likely represent abnormal aggregates of endosomal membranes, somewhat resembling those seen in mammalian cells after over-expression of CHMP4 (mammalian Vps32) paralogues C-terminally tagged with GFP (Katoh et al., 2003; Lin et al., 2005). These results are consistent with the prediction that the fusion protein would be recruited to, but cannot dissociate from endosomal membranes. Using non-inducing conditions we observed Vps32-GFP in motile early endosomes (data not shown) and smaller static aggregates of membranes, as reported (Galindo et al., 2007). In agreement with the contention that these aggregates contain endocytosed membranes, we demonstrated that they are labeled with FM4-64 in uptake experiments (Fig. 4B). These experiments also confirmed that aggregates are associated with vacuolar membranes, often localizing to the junctions between adjacent vacuoles (Fig. 4C).

**Fig. 5:**
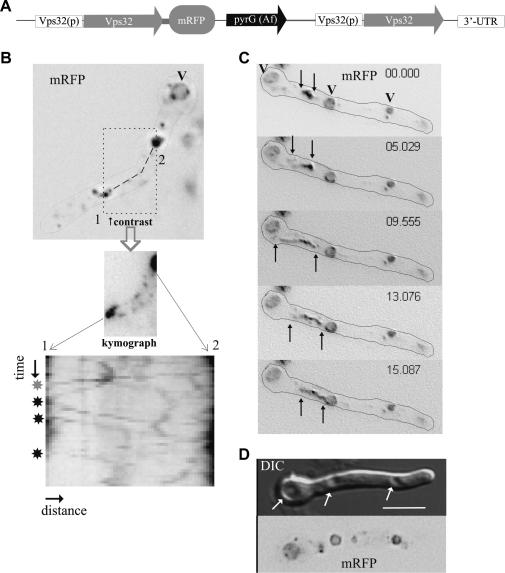
Subcellular localization of Vps32-mRFP using physiological levels of expression. (A) A plasmid encoding Vps32-mRFP expressed under the control of the *vps32* promoter was targeted to the *vps32* locus by homologous integration as described in materials and methods. (B) Localization of Vps32-mRFP: The fluorescent protein localizes to the rim of the vacuoles (v), to relatively static cytosolic aggregates and to bidirectionally motile early endosomes, which are clearly seen in the region located between the two prominent aggregates, numbered 1 and 2, after adjusting the contrast of the image. Time lapse imaging revealed the bidirectional motility of these early endosomes as illustrated by the kymograph displayed at the bottom, which corresponds to a 22 s movie (0.5 s between frames) and an 8 pixel line traced between aggregates 1 and 2. Moving endosomes are seen as diagonals. The three black stars indicate basipetally moving endosomes whereas the grey one indicates one moving acropetally. Relatively static endosomes are seen as approximately vertical lines. This panel should be consulted together with Supplementary movie 1. The large image was treated with the unsharpening mask of METAMORPH, to improve contrast (C). A tubular structure originating from a membrane aggregate located between two vacuoles whose membrane is also labeled with Vps32-mRFP. Note how the tubular structure is extended in the first three frames and shrinks in the last two, apparently connecting with the more acropetal vacuole of the pair. Time is shown in sec. msec. Frames were taken from Supplementary movie 2. (D) Three vacuoles, seen as indentations in the Nomarski image (DIC), showing Vps32-mRFP staining of their membranes. Bar, 5 μm.

We next used a strain that synthesizes Vps32-mRFP under the control of the *vps32^p^* promoter (thus predictably expressing levels of fusion protein similar to physiological levels of endogenous Vps32). This strain contains a single copy of the transgene integrated by homologous recombination into the *vps32* locus, such that *vps32* and *vps32::mRFP* are physically linked (Fig. 5A). This experimental design takes into account the possibility that integration elsewhere into the genome might give rise to position-dependent changes in gene expression and allows co-segregation of wild-type and mRFP tagged *vps32* genes in crosses. No significant growth defects resulting from *vps32::mRFP* expression were noted. The transgene drives the synthesis of a fusion protein of the expected size, as determined by western blot (data not shown). Vps32-mRFP expressed at physiological levels localized to several structures: (i) bright cytosolic specks likely representing late endosomes because they are static. These specks (Fig. 5B) were highly fluorescent, in agreement with the role attributed to Vps32 as the major structural component of ESCRT-III lattices (Teis et al., 2008) and were often adjacent to large or small vacuoles (not shown)(two specks are seen closely associated to the basal vacuole in Fig. 5B); (ii) the membranes of vacuoles themselves (Fig. 5B–D); and (iii) faintly labeled structures that are bidirectionally motile, as clearly seen in kymographs of regions located between the intense, static structures (Fig. 5B; Supplementary movie 1 should be consulted). Taken together, all these data indicate that Vps32 polymers are present on early and late endosomes and that Vps32-mRFP does not undergo normal dissociation from endosomes, which explains why a proportion reaches the vacuolar membrane. In addition, we observed in time lapse movies that Vps32-mRFP decorates tubular structures that transiently connect cytosolic aggregates with vacuoles and undergo extension and subsequent shortening (Fig. 5C and Supplementary movie 2). Similar tubular structures are often detected between vacuoles (our unpublished observations with several vacuolar markers) and have been reported in *Aspergillus oryzae* (Ohneda et al., 2002).

### 3.4 The subcellular localization of DidB^Did2^

We constructed *didB-gfp* strains by gene replacement, such that the fusion protein, expressed at physiological levels, was the only source of DidB. These strains showed a minor impairment of growth, which suggested that GFP C-terminal attachment, impairs function. In agreement, growth tests showed that *didB-gfp* strains phenotypically resemble the null mutant (Fig. 2A). We anticipated this result because, as noted above, fusion of GFP to ESCRT-III proteins disrupts their function in MVB sorting (Nickerson et al., 2006) and leads to dominant-negative effects (Howard et al., 2001). However, as for mammalian Vps32 (Lin et al., 2005), the N-terminal region of *S. cerevisiae* Did2p is necessary and sufficient for its localization to endosomes and C-terminal attachment of GFP to full-length Did2p does not interfere with this localization (Nickerson et al., 2006). Thus, we exploited DidB-GFP to determine the location of ESCRT-III-containing endosomes.

**Fig. 6:**
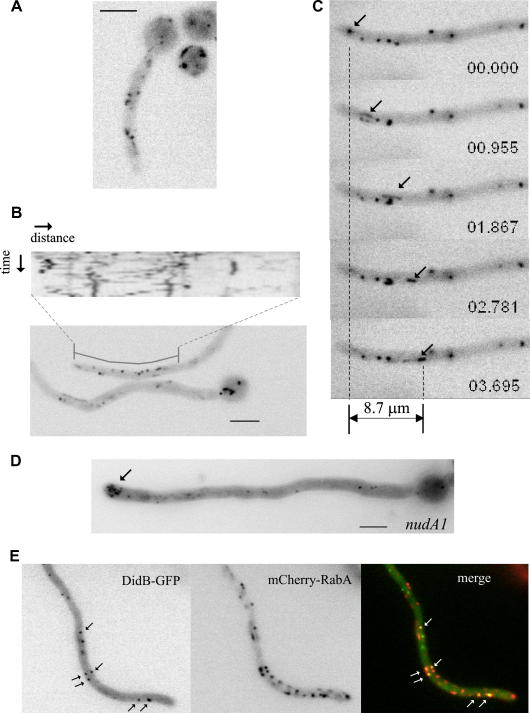
Localization of DidB-GFP expressed at physiological levels. (A) Germling carrying the *didB-gfp* gene replacement. DidB-GFP localizes to cytosolic specks. Time lapse imaging (Supplementary movies 3) revealed that a proportion of these cytosolic specks showed bidirectional movement characteristic of early endosomes. Bar, 5 μm. (B) A hyphal tip cell showing relatively static (vertical lines in the kymograph), probably late endosomes and motile (diagonals in the kymograph) early endosomes. The kymograph corresponds to an 8 pixel-wide and 20 μm-long line (position indicated) over a 20 s time period. Bar, 5 μm. (C) An example of an early endosome (arrow) which moves away from the tip during the indicated time (in sec. msec.). Note its comet tail-like shape due to the relatively long exposure times used to photograph this sequence. (D) Example of *nudA1* cell expressing DidB-GFP. Cells were cultured at 25 °C because the accumulation of endosomes near the tip resulting from the mutation is conspicuous even at this temperature. The picture is a maximum intensity projection of a z-stack of eight images (z-shift, 0.25 μm). Bar, 5 μm. (E) Colocalization of DidB and RabA. Example of a cell co-expressing DidB-GFP and mCherry-RabA (a marker of early endosomes) that was simultaneously photographed in the green and red channels using a DualView beam splitter. Arrows indicate colocalization.

Time-lapse epifluorescence microscopy showed that DidB-GFP localizes to fluorescent cytosolic specks (Fig. 6A) (Supplementary movies 3 and 4). A fraction of these DidB-GFP-containing specks were static or showed short range motility. In FM4-64 time-course experiments (our unpublished data), the endocytic pathway tracer labels motile early endosomes before reaching a class of relatively static non-vacuolar structures that, accordingly, almost certainly represent late endosomes. Thus we hypothesize that the relatively static and brighter DidB-GFP specks are late endosomes. A proportion of DidB-GFP specks, generally the faintest, showed long-range motility, as clearly illustrated in the kymograph shown in Fig. 6B (readers should consult Supplementary movies 3 and 4 to fully appreciate the motility of these specks). In the example shown in Fig. 6C, one fluorescent structure (arrowed) underwent retrograde movement at a velocity >2 μm/s. As such long-range motility defines early endosomes, as a proportion of Vps32-GFP also localizes to motile structures and as DidB is one of the latest-acting ESCRT-III associated proteins (Did2p is recruited to endosomes by Vps24p, the capping factor of polymeric Vps32p structures) (Nickerson et al., 2006), these data strongly indicate that ESCRT-III polymers can assemble on endosomal membranes from the initial steps of the endocytic pathway, at the stage of early endosomes.

To buttress this conclusion, we used *nudA1^ts^*, a conditional mutation in the gene encoding the cytoplasmic dynein heavy chain NudA (Xiang et al., 1995). As endosomes characteristically traffic through the tip region before undergoing dynein-mediated retrograde movement, they accumulate in the tip in a dynein-deficient mutant (Zekert and Fischer, 2008; Abenza et al., 2009). DidB-GFP specks, which in the wild-type are scattered across the hyphae, concentrate at the tip region in the mutant and thus display yet another characteristic of early endosomes (Fig. 6D). However, in contrast to the large aggregates seen in *nudA1* tips with GFP-tagged early endosomal RabA (Abenza et al., 2009), DidB-GFP fluorescence can be resolved into discrete spots, suggesting that only a proportion of endosomes with ‘early’ identity contain the reporter. Dual View colocalization experiments with mCherry-RabA [RabA is an early endosomal Rab5 (Abenza et al., 2009)] supported this conclusion: RabA structures are clearly more abundant than DidB structures, but DidB-GFP and mCherry-RabA colocalize (Fig. 6E). However, some DidB-GFP structures do not contain RabA (Fig. 6E), suggesting that DidB predominates in RabA-containing early endosomes but it is also present in other endosomes lacking RabA. Maturation of fungal early endosomes into late endosomes involves conversion of Rab5 domains into Rab7 domains (Peplowska et al., 2007). Thus we speculate that the minor proportion of DidB-positive endosomes that do not contain RabA are late endosomes that have undergone such conversion.

### 3.5 Synthetic interaction of *didBΔ* and *alcA^p^*::*vps32-gfp*

**Fig. 7:**
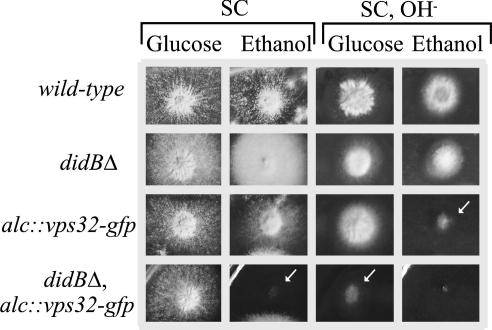
Vps32-GFP over-expression interacts synthetically with *didBΔ*. Strains with the indicated relevant genotypes were cultured on SC containing 1% ethanol or 3% glucose as sole carbon source (inducing and repressing conditions for *alcA^p^*, respectively), adjusted to alkaline pH when indicated (OH^−^). Arrows indicate informative combinations.

The fact that C-terminal attachment of GFP impairs Vps32 function led us to hypothesize that Vps32-GFP over-expression might result in negative-dominant effects. Fig. 7 shows that this is indeed the case. Vps32-GFP over-expression (ethanol, inducing conditions) slightly impairs growth on synthetic neutral pH medium and nearly abolishes it on alkaline medium (Fig. 7, second and fourth columns, respectively). As deletion of *S. cerevisiae* genes involved in vacuolar acidification/biogenesis precludes growth at alkaline pH (Serrano et al., 2004) and Vps32-GFP over-expression results in large aggregates of endosomal membranes (Fig. 4A), we hypothesized that both the growth impairment and the alkaline pH sensitivity caused by Vps32-GFP over-expression result from dysfunction of the endosomal system. Indeed, growth tests demonstrated that Vps32-GFP over-expression is synthetically lethal with *didBΔ* (Fig. 7, second column): the double mutant hardly grows under alkaline conditions even when Vps32-GFP levels are very low because expression of the transgene is repressed (Fig. 7, third row) (repression does not completely abolish *alcA^p^*-driven expression). The fact that the absence of DidB exacerbates the growth defect resulting from Vps32-GFP over-expression would be consistent with the inability of the fusion protein to undergo dissociation from the plasma membrane, as reported for *S. cerevisiae*.

## 4 Discussion

We report here the genetic characterization of *didB*, a non-essential *A. nidulans* gene of the multivesicular body pathway. The *S. cerevisiae* and human orthologues of DidB, denoted Did2p and CHMP1A/CHMP1B, respectively, interact directly with Vps4, the AAA ATPase that mediates disassembly of ESCRT complexes from endosomal membranes. Using bacterially expressed proteins, we demonstrate that the ability to interact directly with Vps4 is conserved in DidB.

Despite the fact that the MVB role of Did2p involves its interaction with ESCRT-III and Vps4p, its precise molecular role is not yet clear, as the major recruiter of Vps4p to ESCRT-III polymers is Vps2p (Obita et al., 2007; Saksena et al., 2009; Wollert et al., 2009) Current models favor the view that Did2, in complex with Ist1, regulates an interaction between ESCRT-III and Vps4 that favors the release of ESCRT-III subunits from the polymer, in agreement with the inability of *S. cerevisiae did2Δ* mutants to dissociate efficiently ESCRT-III from endosomal membranes (Nickerson et al., 2006; Rue et al., 2008). However, this impairment does not preclude the inward budding of vesicles into the endosomal lumen (Nickerson et al., 2006) and leads to a weak MVB sorting phenotype compared to the complete block resulting from the absence of Vps4p (Rue et al., 2008).

We have previously shown that the Vps2-Vps4 interaction is conserved in *A. nidulans* (Galindo et al., 2007), suggesting that, like its budding yeast orthologue, DidB would not play an essential Vps4-recruiting role in the MVB pathway. In contrast to deletion mutants in key ESCRT components (Calcagno-Pizarelli, A.M., M.A.P and H.N.A., unpublished) (Rodríguez-Galán et al., 2009) and in agreement with this prediction, *didB*Δ mutants are viable and able to deliver the endocytic cargo AgtA to the vacuolar lumen, demonstrating that *didB*Δ does not block the MVB pathway. However, they show reduced turnover of this transporter, correlating with its persistent presence at the plasma membrane and thus showing that *didB*Δ leads to a detectable trafficking phenotype. Indeed, *didB*Δ mutants show impaired growth at alkaline pH, a phenotype consistent with a partially defective endosomal system. We further demonstrate that over-expression of Vps32-GFP leads to a dominant-negative growth phenotype, in agreement with the prediction that C-terminal GFP attachment precludes Vps32 disassembly from ESCRT-III polymers. This phenotype is largely exacerbated by *didBΔ*, as would be expected if DidB contributes to the disassembly of ESCRT-III.

In previous work (Galindo et al., 2007) we reported, in the context of our analysis of the pH signaling cascade, that Vps32 localizes, in part, to early endosomes, which in *A nidulans* and *Ustilago maydis* show distinctive long-range bidirectional motility (Lenz et al., 2006; Abenza et al., 2009). However, we had not yet published our detailed observations on the subcellular localization of ESCRT-III and report them here. Vps32-GFP and Vps32-mRFP localize, in addition to early endosomes, to static, possibly ‘late’ endosomes, to the vacuolar membrane and to membrane aggregates adjacent to the vacuoles which become more prominent with increasing levels of Vps32 expression. These localizations almost certainly reflect the inability of these proteins to disassemble from endosomal membranes, thus trafficking with them to reach the last compartments in the endocytic pathway. These observations were combined with experiments using endogenously tagged DidB-GFP. While DidB-GFP is also present in static structures likely representing late endosomes, a proportion of the reporter localizes to early endosomes as shown by their characteristic rapid, long-distance movement and their colocalization with the early endosomal marker RabA. Moreover, like early endosomes, discrete structures containing DidB-GFP accumulate in the tip region in a dynein-deficient mutant. This almost certainly reflects the fact that a proportion of early endosomal membranes that coalesce in the mutant tip region can recruit ESCRT-III.

DidB is a ‘late’ ESCRT-III protein whose recruitment to the complex is Vps24-mediated (Nickerson et al., 2006). Thus these data demonstrate that ESCRT-III assembly on fungal endosomal membranes can take place at the level of early endosomes, in agreement with the finding that ESCRT-0 recruitment and ubiquitin-mediated sorting into the MVB pathway occur in mammalian early endosomes (Raiborg et al., 2002).

## Figures and Tables

**Table 1:** Strains used in this work.

MAD1017	*biA1; pantoB100*	Our collection
MAD1090	*palA1; pantoB100*	Our collection
MAD1315	*wA2; inoB2; paC900*	Our collection
MAD1471	*yA2; argB2 [argB∗::alcA^p^::vps32::gfp]; pantoB100*	Galindo et al. (2007)
MAD1598	*argB2; pyroA4; nkuA::argB^Af^ riboB2 didBΔ::pyroA^Af^*	This work
MAD1630	*pabaA1; wA2; pyroA4; didBΔ::pyroA^Af^*	This work
MAD1717	*wA2; pyroA4; pacC900; didBΔ::pyroA^Af^*	This work
MAD1764	*yA2pabaA1 agtA::gfp::*:*pyrG^Af^ pyrG89; pyroA4*	Our collection
MAD1851	*wA2; pyroA4; pantoB100; didBΔ::pyroA^Af^*	This work
MAD1879	*pabaA1agtA::gfp::pyrG^Af^* (*pyrG89?*)*;pyroA4; didBΔ::pyroA^Af^*	This work
MAD1880	*agtA::gfp::pyrG^Af^* (*pyrG89?*)*; pyroA4;didBΔ::pyroA^Af^*	This work
MAD1883	*pyrG89; pyroA4 ΔnkuA::bar; didB::gfp::pyrG^Af^*	This work
MAD1921	*yA2 pyrG89; vps32::mrfp::pyrG^Af^::vps32 argB2[argB∗::alcA^p^::palC::gfp] inoB2areA^r^18 palC4*	Our collection
MAD1961	*wA2 palH72; inoB2; pacC900; didBΔ::pyroA^Af^*	This work
MAD1962	*wA2 palH72; inoB2; pacC900*	This work
MAD1964	*yA2; argB2 [argB∗::alcA^p^::vps32::gfp]*	This work
MAD2876	*pyrG89?; ΔnkuA::bar; didB::gfp::pyrG^Af^ nudA1*	This work
MAD2878	*pyrG89?; ΔnkuA::bar; pantoB100; didB::gfp::pyrG^Af^*	This work
MAD2879	*pyrG89?; argB2[argB∗::alcA^p^::mCherry::rabA]; didB::gfp:: pyrG^Af^*	This work

## Appendix A Supplementary material

Supplementary movie 1 Time-lapse movie of endosomes labeled with Vps32-mRFP expressed at physiological levels. Bidirectionally moving early endosomes seen together with large, static membrane aggregates (over-contrasted to reveal fainter early endosomes). Time is in sec. msec.

Supplementary movie 2 A tubular structure decorated with Vps32-mRFP localized between two vacuoles, whose membrane is also decorated with the fusion protein. Time is in sec. msec.

Supplementary movie 3 Short germling carrying the *didB::gfp* gene replacement allele. Time is in sec. msec. Bar, 5 μm.

Supplementary movie 4 Dynamics of DidB-GFP endosomes in a hyphal tip cell (top). Time is in sec. msec.
