# Supplementary figures and images for: Characterization of Aspergillus nidulans DidBDid2, a non-essential component of the multivesicular body pathway
Source: Fungal Genet Biol. 2010 Jul;47(7):636–46. doi: 10.1016/j.fgb.2010.03.010 (PMC2884189; doi:10.1016/j.fgb.2010.03.010)

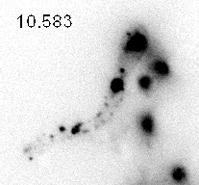

Supplement: Supplementary movie 1 — Time-lapse movie of endosomes labeled with Vps32-mRFP expressed at physiological levels. Bidirectionally moving early endosomes seen together with large, static membrane aggregates (over-contrasted to reveal fainter early endosomes). Time is in sec. msec. [file mmc1.jpg]

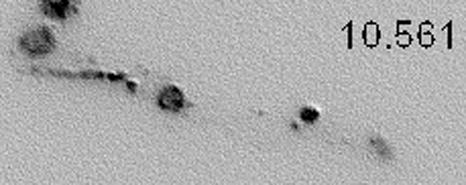

Supplement: Supplementary movie 2 — A tubular structure decorated with Vps32-mRFP localized between two vacuoles, whose membrane is also decorated with the fusion protein. Time is in sec. msec. [file mmc2.jpg]

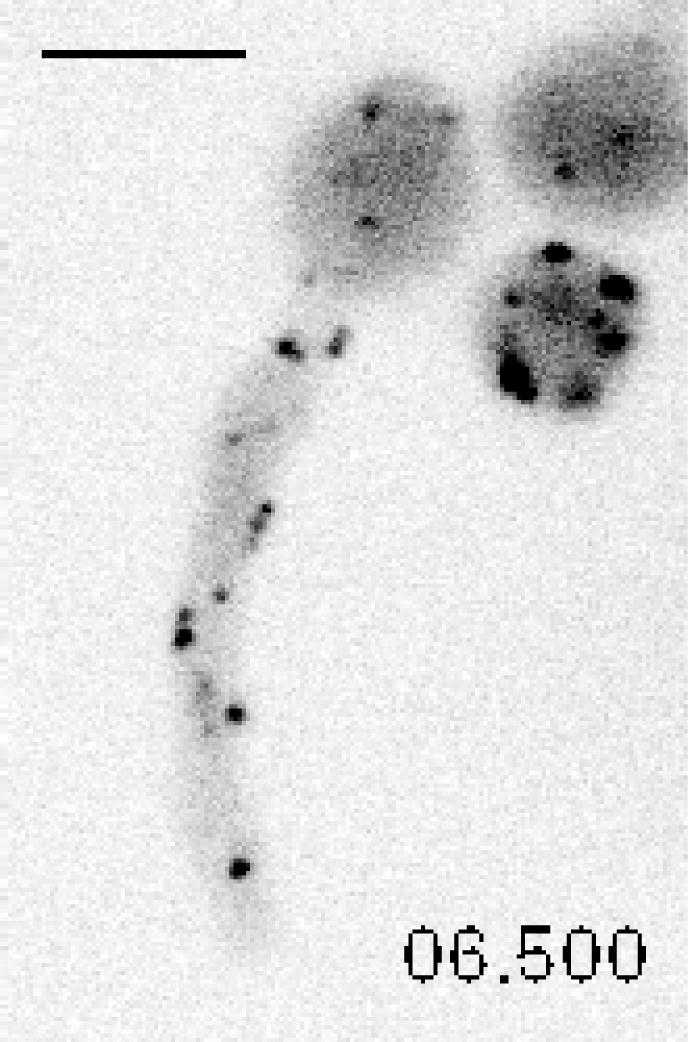

Supplement: Supplementary movie 3 — Short germling carrying the didB::gfp gene replacement allele. Time is in sec. msec. Bar, 5 μm. [file mmc3.jpg]

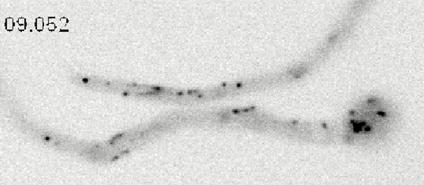

Supplement: Supplementary movie 4 — Dynamics of DidB-GFP endosomes in a hyphal tip cell (top). Time is in sec. msec. [file mmc4.jpg]
